# KangFuXin Liquid in the Treatment of Diabetic Foot Ulcer: A Systematic Review and Meta-Analysis

**DOI:** 10.1155/2019/3678714

**Published:** 2019-12-30

**Authors:** Ke Shen Qu, Yang Li, Yue Liang, Xiao Jie Hu, Xuan Yu Wang, Xin Chen, Hua Fa Que

**Affiliations:** ^1^Department of Traditional Chinese Surgery, Longhua Hospital, Shanghai University of Traditional Chinese Medicine, Shanghai 200032, China; ^2^Shanghai University of Traditional Chinese Medicine, Shanghai 201203, China

## Abstract

**Background:**

Diabetic foot ulcer (DFU) is one of the most common complications of diabetes mellitus, with the wound not healing as expected and healing slowly. Poor control can develop into gangrene and even amputation. Currently, the existing treatments are not satisfactory enough. In China, KangFuXin liquid (KFXL) has been clinically used to treat DFU and has shown good clinical efficacy. In order to provide more reference to clinicians and experts, evidence of efficacy for it needs to be further rigorously evaluated.

**Methods:**

Eight electronic databases were searched to identify eligible randomized clinical trials (RCTs) published from construction of the library to April 2019. There is no language or data restriction; 11 trials involving 889 participants met the inclusion criteria. These RCTs compared the total effective rate, cure rate, cure time, and adverse events associated with KFXL. The Cochrane Handbook guidelines were used to assess the risk of bias and to evaluate the methodological quality of eligible studies. The methodological quality of included studies was generally low. Dichotomous and continuous data were presented using risk ratios (RRs) and mean differences (MDs), respectively.

**Results:**

Compared with the basic treatment, meta-analyses showed that KFXL combined with basic treatment can improve the total effective rate (RR = 1.38; 95% CI = 1.23–1.54; *P* < 0.00001; fixed effect model: *I*^2^ = 32%) and cure rate (RR = 1.67; 95% CI = 1.17–2.38; *P*=0.005; random effect model: *I*^2^ = 65%), and shorten the healing time (MD = −5.73; 95% CI = −6.95 to −4.52; *P* < 0.00001; random effect model). Moreover, under the same basic treatment, KFXL had a better effect than external use of pharmaceutical medications (RR = 1.95; 95% CI = 1.30–2.93; *P*=0.001), but the cure rate was not significantly different. Also, KFXL had nothing to do with adverse reactions.

**Conclusion:**

The evidence confirms that KFXL is an effective treatment for DFU. However, further large-scale, rigorously designed trials and high-quality studies are needed to confirm the role of KFXL in the treatment of DFU.

## 1. Introduction

Diabetic foot ulcer (DFU), which is usually associated with peripheral neuropathy, limb circulatory disturbance, and infection, is one of the most common complications of diabetes mellitus. The incidence rate of DFU is about 4–10% and continuing to grow [1]. Compared with healthy individuals, the repair of wounds in patients with DFU is slow. The wound cannot heal as expected and develop into gangrene or even amputation. Therefore, compared with nondiabetic patients, patients with DFU have higher amputation rates and mortality [2], and amputation rates account for 70% of nontraumatic amputations worldwide [3], which brings severe economic stress and mental burden to them [4]. According to the “Guidelines for the diagnosis and treatment for diabetic ulcer/gangrene,” the recommended treatment for DFU mainly includes glucose-level control, anti-infection, surgical debridement, and external use of growth factor [1]. However, the effectiveness of the recommended treatment is not satisfying enough. Even with a comprehensive treatment regimen, the cure rate of DFU at 12 to 20 weeks is as low as 24% to 30% [5, 6], and even if the ulcer heals after treatment, the risk of recurrence is as high as ten times [7].

Given the above, more effective treatment options should be considered. KFXL, a pure Chinese herbal medicine extracted from the *Periplaneta americana*, has been widely used to treat various ulcerative diseases, especially skin ulcers. The *Periplaneta americana* was first recorded in the Han Dynasty ancient books named “*Shen Nong's Herbal Classic*,” which has the effect of breaking through the phlegm, resolving to accumulate, reducing swelling, detoxify, and activating blood to resolve stasis [8]. It has been used for thousands of years to treat snake bites, skin ulcers, and burns. Pharmacological research shows KFXL can enhance immunity, reduce inflammation, promote epidermal cell growth, granulation tissue proliferation to repair the damage, and accelerating the repair and regeneration of damaged tissue to promote wound healing [9–15]. In China, this pure traditional Chinese medicine extract has been widely used in the clinical treatment of DFU. The clinical efficacy of KFXL has been reported in some clinical studies [16].

However, there is currently no systematic review to fully evaluate the clinical evidence for the treatment of DFU with KFXL. Therefore, we evaluated the effectiveness of KFXL in the treatment of DFU through systematic reviews and meta-analysis.

## 2. Materials and Methods

Before initiating the review process, the review protocol was registered in the PROSPERO database (CRD42019131516). We performed this study according to the Cochrane Handbook for Systematic Reviews of Interventions and followed the instruction of Preferred Reporting Items for Systematic Reviews and Meta-analyses (PRISMA) guidelines.

### 2.1. Data Search Strategies

Two reviewers used the search terms “KangFuXin solution,” “KangFuXin liquid,” “diabetic foot,” “diabetic foot ulcer” to systematically search for relevant randomized clinical trials (RCTs) in PubMed, EMBASE, Cochrane Central Register of Controlled Trials (CENTRAL), China National Knowledge Infrastructure (CNKI), WanFang Database, Chinese Biomedical Literature Database (CBM), Chinese Scientific Journals Database (VIP), and Google Scholar. The search time limit was between the construction of the library to April of 2019. There was no restriction on the language and the type of publication, including proceedings, postgraduate theses, and papers with abstracts only.

### 2.2. Inclusion Criteria

#### 2.2.1. Types of Studies

We selected all the RCTs for meta-analysis. Quasi-RCTs, non-RCTs, and randomized trials with false randomization methods were excluded.

#### 2.2.2. Participants

Those diagnosed with DFU by the World Health Organization (WHO), International Federation of Vascular Diseases (IUA), Chinese Medical Association (CMA), or other authoritative diagnostic criteria, regardless of the age, gender, nationality, and ethnicity.

#### 2.2.3. Intervention

The experimental group used KFXL combined with basic treatment (basic internal medical treatment and conventional debridement). The administration method of KFXL was not limited (wet application, spraying, or rinsing), and the dressing is not limited (sterile dry gauze or vaseline gauze). Basic internal medical treatments included blood glucose control and anti-infective, and the administration methods are oral, intravenous infusion, and subcutaneous administration; Conventional debridement included saline or hydrogen peroxide to flush wound secretions, iodophor disinfection, and surgical debridement to remove necrotic tissue.

#### 2.2.4. Control Group

The control group was patient with DFU who was not treated with KFXL only received basic treatment.

#### 2.2.5. Outcome Measures

The primary outcome was the total effective rate during treatment, defined by changes in the size of the wound ulcer. The calculation formula is as follows: total effective rate = cure rate + effective rate. We also evaluated the cure rate and cure time during treatment, defined by the complete healing of the ulcer, as well as adverse events.

The study will be excluded if any of the following is true: 1. The original data of the literature was incomplete or erroneous, the effective rate was unclear, and the data cannot be extracted, resulting in the inability to assess the primary or secondary outcome 2. Animal experiments, case reports, literature reviews, and systematic reviews; 3. The experimental group had oral or external interventions for other traditional Chinese medicine preparations other than KFXL.

### 2.3. Data Extraction

Two authors (Ke Shen Qu, Xiao Jie Hu) extracted basic information independently according to the before mentioned inclusion criteria. Further discussions by the third author (Hua Fa Que) helped resolve the disagreement. The data extracted from the enrolled literature included the following: first author's name, publication time, literature source, diagnostic criteria, a sample size of the experimental group and control group, intervention measures, intervention time, frequency of medication, outcome index, adverse events, and manufacturer of KFXL.

### 2.4. Risk of Bias Assessment

Each of the included RCTs needs to be assessed for risk of bias, which was done independently by the two author (Xin Chen, Xuan Yu Wang) using the Cochrane Risk of bias tool [17], and the disputed part resolves the disagreement through negotiation or a third author (Hua Fa Que).

### 2.5. Statistical Analysis

We performed this meta-analysis using Revman 5.3 software (Cochrane Collaboration) [18] for all statistical data analyses, using 95% confidence interval (CI) and risk ratio (RR) to calculate categorical variables, and using 95% CI and mean differences (MDs) to calculate continuous variables. Statistical heterogeneity was tested for included trials. If the trial had acceptable homogeneity (*I*^2^ < 85%) in participants, study design, controls, interventions, and outcome measures, a meta-analysis were performed. The fixed effect model (*I*^2^ < 25%) was used for homogeneous studies, and the random effects method was used for studies with substantial heterogeneity before the fixed effect model (25% < *I*^2^ < 85%) [19].

## 3. Result

### 3.1. Database Search

**Figure 1:**
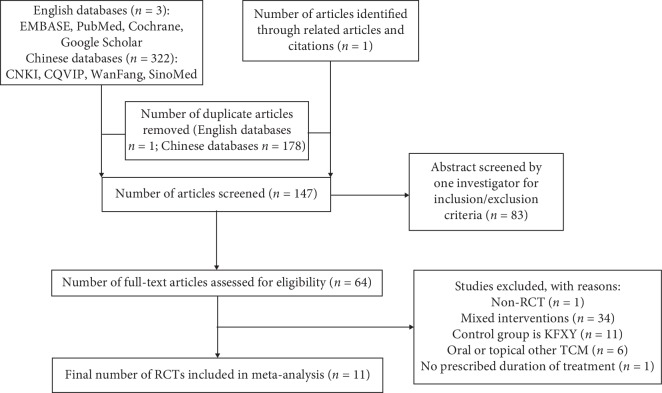
Summary of the literature identification and selection process.

After searching eight databases, 326 studies were identified. Among them, 179 studies were excluded because they did not meet the inclusion criteria. The full text of 64 studies was assessed for eligibility. Among them, 53 studies were excluded for the following reasons: were mixed interventions (*n* = 34), control groups were KFXL (*n* = 11), use of oral or topical other Chinese medicines (*n* = 6); non-RCT study (*n* = 1), and no clear treatment time (*n* = 1). Ultimately, a total of 11 studies [20–30] were included in this systematic review and meta-analysis (see Figure 1). All studies were published in Chinese. The characteristics of the included studies are illustrated in Tables 1 and 2.

### 3.2. Study Characteristics

Eleven studies [20–30] were included in the study, with a total of 889 participants, 449 and 440 in the experimental and control groups, respectively. The sample size of these trials ranged from 10 to 67, and 1 study [22] reported adverse events. KFXL used in each study was from several different manufacturers, but the ingredients were ethanol extracts of the *American cockroach*.

### 3.3. Risk of Bias Assessment

**Figure 2:**
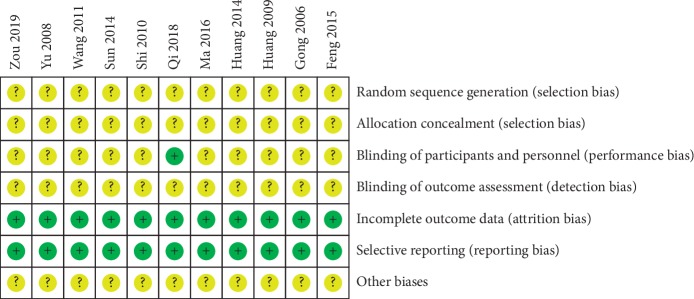
Risk of bias graph.

The details of the risk of bias of each study are shown in Figure 2; the literature included in our study is poor in methodological quality. All of the studies used the principle of randomization, and none of them described the specific method. Also, only 1 study [22] reported the blinded information to participants and researchers. No studies reported withdrawals and dropout numbers. Selective reporting was fully addressed in all studies. We found no other biases in these studies. We determined that other sources of bias were assessed as unclear risk of bias in all of the studies. Given the poor methodological quality, we recommend that research methods and sample representation should be improved in future studies.

### 3.4. Primary Outcomes

#### 3.4.1. Total effective Rate of KFXL Combined with Basic Treatment versus Basic Treatment

**Figure 3:**
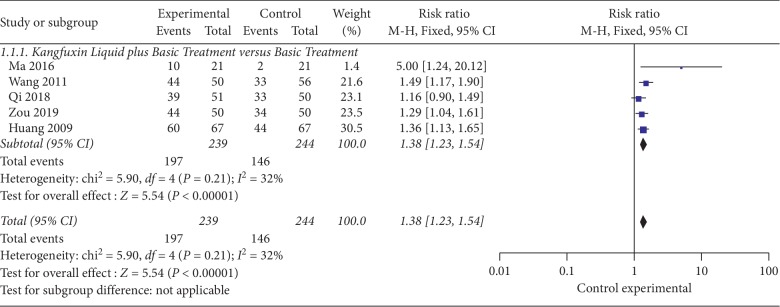
The total effective rate of KFXL combined with basic treatment versus basic treatment.

The 5 RCTs [20–24] contained 483 patients; The experimental group was KFXL combined with basic treatment, and the control group was basic treatment alone. Using the fixed effect model, analysis showed a significant difference in the total effective rate of the KFXL combined with basic treatment compared with the basic treatment alone (RR = 1.38; 95% CI, 1.23–1.54; *P* < 0.00001; *I*^2^ = 32%) (see Figure 3).

#### 3.4.2. Total effective Rate of KFXL versus External Use of Pharmaceutical Medications Based on Same Basic Treatment

**Figure 4:**
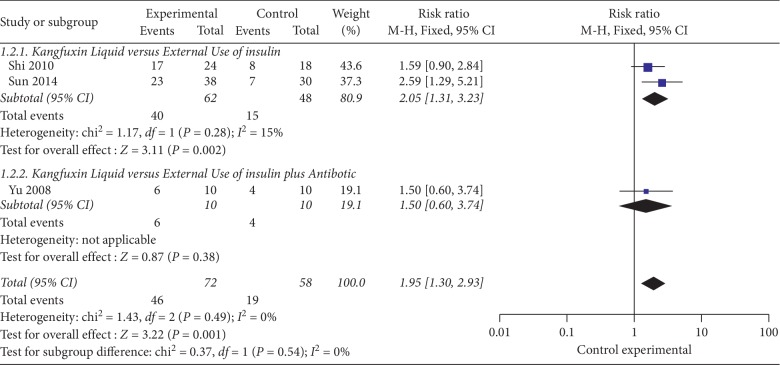
The total effective rate KFXL versus external use of pharmaceutical medications based on same basic treatment.

The 3 RCTs [25–27] contained 130 patients. Under same basic treatment, the total effective rate between KFXL and external use of pharmaceutical medications was compared. Using the fixed effect model analysis results, the total effective rate of KFXL showed a significant difference compared with the external use of pharmaceutical medications (RR = 1.95; 95% CI, 1.30–2.93; *P*=0.001; *I*^2^ = 15%) (see Figure 4).

Based on different use of pharmaceutical medications, the control group can be divided into two different subgroups: external use insulin alone and external use insulin combined with antibiotic. Two trials [25, 26] compared the KFXL and external use of insulin, and the results showed that there was a significant difference in KFXL compared with external use of insulin (RR = 2.05; 95% CI, 1.3–3.23; *P*=0.002); 1 trial [27] compared the KFXL and external use of insulin combined with antibiotics. However, the results showed that there was no difference in the total effective rate (RR = 1.50; 95% CI, 0.60–3.74; *P*=0.38).

#### 3.4.3. Total Effective Rate of KFXL Combined with Insulin versus External Use of Insulin Based on Same Basic Treatment

1 trial [28] compared external use of KFXL combined with insulin and external use of insulin based on same basic treatment. The results showed that there was a significant difference between external use of KFXL combined with insulin (RR = 1.30; 95% CI, 1.01–1.68; *P*=0.04).

### 3.5. Secondary Outcomes

#### 3.5.1. Cure Rate

**Figure 5:**
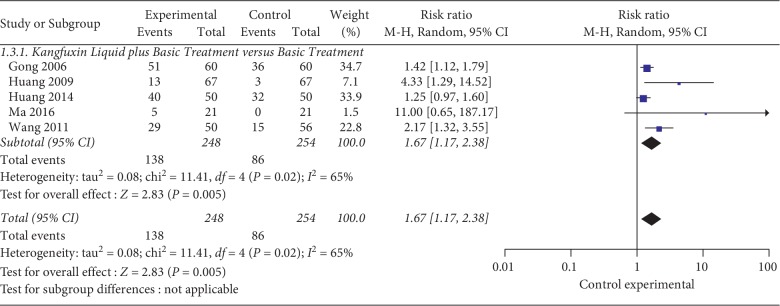
Meta-analysis of the cure rate of KFXL combined with basic treatment. versus basic treatment.

1. Cure rate of KFXL combined with basic treatment versus basic treatment: 5 trials [20, 21, 23, 29, 30] containing 502 cases was reported the cure rate. The experimental group was treated with basic treatment combined with KFXL, and the control group was treated with basic treatment. Using the random effect model, the results of the analysis showed that there was a significant difference between basic treatment combined with KFXL compared with basic treatment alone (RR = 1.67; 95% CI, 1.17–2.38; *P*=0.005; *I*^2^ = 65%) (see Figure 5).
2. Cure rate of KFXL combined with insulin versus external use of insulin based on same basic treatment: 1 trial [25] compared external use of KFXL and insulin under same basic treatment. The results showed that there was no significant difference between KFXL and external use of insulin (RR = 2.25; 95% CI, 0.51–9.87; *P*=0.28).

#### 3.5.2. Cure Time

**Figure 6:**
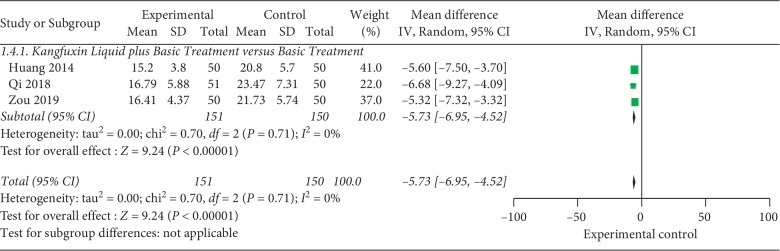
Cure time of KFXL combined with basic treatment versus basic treatment.

1. Cure time of KFXL combined with basic treatment versus basic treatment: 3 trials [22, 24, 29] containing 301 cases reported the rate of cure time. The experimental group was treated with KFXL under basic treatment, and the control group was treated with basic treatment alone. Using the random effect model, the results showed that the cure time of KFXL combined with basic treatment was significantly lower than basic treatment alone (MD = −5.73; 95% CI, −6.95 to −4.52; *P* < 0.00001) (see Figure 6).
2. Cure time of KFXL and external use of pharmaceutical medications based on same basic treatment: 1 trial [27] compared the KFXL and insulin combined with antibiotic based on basic treatment. The results showed that the two therapies had differences in healing time (MD = −4.70; 95% CI, −8.30 to −1.10; *P*=0.01).

### 3.6. Adverse Events

Only 1 trial [22] reported adverse events during treatment in 11 trials included, describing the incidence of adverse events in the experimental and control groups. Adverse events mainly manifested as headache, dizziness, palpitations, and dysfunction of liver and kidney, caused by basic internal medical treatment—oral cilostazol—the contrast was not statistically significant. The remaining trials did not describe the occurrence of adverse reactions during the use of KFXL.

## 4. Discussion

### 4.1. Summary of Outcomes

We finally included 11 RCTs involving 889 patients after extraction. They used an ethanol extract of *Periplaneta americana*, collectively known as KFXL, to intervene in DFU patients and judge their clinical effects, even though they came from different manufacturers but had the same ingredients. We did the meta-analysis based on that.

Compared with the basic treatment, KFXL combined with basic treatment can improve the total effective rate, healing rate, and shorten the healing time. Through subgroup analysis results, under the same basic treatment, KFXL was compared with the external use of insulin, the former has better efficiency than the latter. Other than this, KFXL compared with external use of insulin combined with antibiotics can promote wound cure time but has no effect on total effective rate. The above results may represent that the main mechanism of KFXL in the treatment of DFU may be mainly based on repair and promotion of healing. 1 trial [26] also reported the effects of KFXL on transcutaneous oxygen pressure and flowing velocity of dorsal foot blood. After the treatment, the transcutaneous oxygen pressure and the flowing velocity of dorsal foot blood were significantly increased. This suggests that the KFXL may be able to increase the local blood supply, which coincides with the theory of Chinese medicine that can activate blood to resolve stasis.

KFXL is considered to be unrelated to adverse reactions. Adverse effects reported in these trials included headache, dizziness, palpitations, and liver and kidney dysfunction. But statistical analysis was not associated with KFXL and was more likely to be caused by internal medical treatment. Therefore, KFXL is considered to be a better choice for clinical treatment of DFU.

### 4.2. Advantages and Limitations

In the 11 studies we included, no sample loss was reported, and all the outcomes were reported. All the subjects were Chinese, the gender ratio was balanced, and the age was mainly middle aged and elderly. Although this study clarifies the possibility of KFXL as a viable treatment option for DFU, there are still some limitations to this meta-analysis. Within all trials, only 1 trial [22] reported the blinded information to participants and researchers, but the rest trials did not mention the use of blinding. All of the trials are mentioned for grouping using random methods but did not describe specific methods. Therefore, potential performance bias and detection biases were caused by insufficient randomization and lack of blinding. Furthermore, this systematic review and meta-analysis only included 11 trials, and the sample size was small. In addition, there were 6 trials [20, 21, 25–27, 29] that conducted Wagner classification, with the proportions from grade I to grade V being 48%, 36%, 11.3%, 4%, and 0.7% respectively. Moreover, the duration of treatment and dose of the KFXL are not consistent. Therefore, we only recommend that the intervention of KFXL for patients with grade I-II mild to moderate DFU may achieve better results and promote repair in the early stage of ulcer formation to avoid the deterioration of the condition caused by difficult healing of the long-term wound. Only 1 trial [22] reported adverse events, indicating that the safety assessment of KFXL was inadequate, so more research on safety and tolerability is still needed. Last but not least, the clinical study on the use of KFXL for the treatment of DFU lacks a detailed and meticulous design. In addition, the quality of the methodology of this research included in this review was generally poor, indicating that there may be high risk of bias.

### 4.3. Possibility and Rationality of KFXL for the Treatment of DFU

DFU are characterized by slow wound healing. KFXL is rich in active substances such as sex pheromones, proteins, amino acids, affinity peptides, alkaloids, adipokinetic hormones, and polysaccharides, which can reduce inflammatory factor, such as IL-6, IL-8, TNF-a, and c-reactive protein, increase CD8+ T-cell activity and SOD content, increase EGF and VEGF in wound tissue, and TGF-*β* and bFGF levels [31–36]. Recent studies have shown that the mechanism of wound healing from the *Periplaneta americana* extract may be through the regulation of JAK/STAT3, PI3K/AKT, nuclear factor kappa B canonical pathway, and extracellular signal-regulated kinas signaling to affect cell proliferation, fibrogenesis, re-epithelialization, and remodeling [13, 14, 37]. At the same time, the compound periplanosides A-C can stimulate the production of human epidermal fibroblast collagen at a certain concentration [38]. Overall, KFXL, basically an alcohol extract of *Periplaneta americana*, assists in resisting inflammation, diminishing swelling, accelerating the repair of tissue lesions, and enhancing immunity [39].

Complementary and alternative medicine (CAM) is the crystallization of the experience of the Chinese people accumulating in the struggle against diseases. Practitioners of TCM believe that the pathogenesis of DFU is mainly “Qi deficiency and blood stasis.” In the context of TCM, “Qi” can be understood as the general term for the substances, energy, and information that constitute the human body and maintain the life activities of the human body [40]. “Qi deficiency and blood stasis” is a pathological condition. “Qi” can promote the operation of blood. When “Qi” is weak, it is unable to push blood and cause blood stasis. Therefore, whether it is from the perspective of traditional Chinese medicine or modern medicine, KFXL has rationality and possibility in treating DFU.

## 5. Conclusions

Our systematic review and meta-analysis revealed that KFXL could increase the clinical efficacy of basic treatment. Therefore, we recommend that KFXL is suitable for patients with mild to moderate DFU. This article provides new ideas and new methods for better treatment of DFU in the clinic. In the future, more clinical studies should be designed to confirm the effectiveness and safety of KFXL. It can also be combined with other modern treatments for research. However, the overall methodological and reporting quality of the included trials was limited, and more dedicated design and high-quality studies are needed to confirm the role of KFXL in the treatment of DFU. Therefore, more high-quality large–sample size RCTs are required to confirm and explain it.

## Figures and Tables

**Table 1:** Characteristics of 11 included trials.

Study	Intervention	Frequency	Duration of treatment (weeks)	Pharmacological treatment	Main outcomes	Manufacturer
Ma and Ji [21]	KFXL	1	4	Hypoglycemic, antibiotics if necessary	Total effective rate	Good doctor
					Bacterial count	
Huang et al. [29]	KFXL	1	NR	Insulin, antibiotics if necessary, nutritional support	Cure rate	SINOWAY
					Cure time	
Wang and Qu [23]	KFXL	1	3	Insulin, antibiotics, improve blood circulation, nutritional support	Total effective rate	NR
Huang and Lin [20]	KFXL	1-2	4	Insulin, antibiotics	Cure rate	NR
Gong et al. [30]	KFXL	1-2	6	Insulin, antibiotics if necessary	Cure rate	Good doctor
Qi et al. [22]	KFXL	1	2	Vasodilator	Total effective rate	Good doctor
					Cure time	
					Shrinkage rate	
					Nerve conduction velocity	
Feng and Zhao [28]	KFXL plus insulin	1	3	Insulin, improve blood circulation, antibiotics if necessary, nutritional support	Total effective rate	SINOWAY
Shi et al. [25]	KFXL	1	2	Insulin	Total effective rate	Good doctor
					Cure time	
					Total effective rate	
Sun and Gong [26]	KFXL	4	4	Insulin, improve blood circulation, antibiotics if necessary	Oxygen partial pressure blood flow velocity	NR
Yu et al. [27]	KFXL	1	4	Insulin, antibiotics if necessary, nutritional support, vasodilator	Total effective rate	Good doctor
					Cure time	
Zou and Liu [24]	KFXL	1	4	Insulin, antibiotics if necessary	Total effective rate	KELUN
					Cure time	
					Anxiety and depression score	

NR, no report.

**Table 2:** 

Study	Sample		Age		Duration of treatment (years)		Diagnostic criteria	Wagner	Baseline data comparable	Incomplete outcome data	Selective reporting	ADs
	E	C	E	C	E	C						
Ma [21]	21	21	52.4 ± 4.2	51.3 ± 4.0	11.4 ± 3.6	WHO	Yes	Yes	No	No	NR	
Huang [29]	50	50	NR	NR	NR	NR	Yes	Yes	No	No	NR	
Wang and Qu [23]	50	56	57.9 ± 8.9	58.7 ± 7.7	NR	NR	No	Yes	No	No	NR	
Huang [15]	67	67	54	56	NR	WHO1995	Yes	Yes	No	No	NR	
Gong[30]	60	60	69.8		>2		CMA1995	No	Yes	No	No	NR
Qi [22]	51	50	65.1 ± 5.7		7.9 ± 3.1		IUA2013	No	Yes	No	No	Yes (4 patients in the experimental group and 5 patients in the control group reported headache, dizziness, palpitations, and dysfunction of liver and kidney)
Feng and Zhao [28]	28	28	34–70	38–69	NR		Guidelines of type 2 diabetes in China 2007	No	Yes	No	No	NR
Shi [25]	24	18	59.5 ± 9.8	66.5 ± 8.1	1.8 ± 0.5	1.7 ± 0.5	ADA	Yes	Yes	No	No	NR
Sun and Gong [26]	38	30	60.2 ± 5.5		11.4 ± 3.6		WHO 2007	Yes	Yes	No	No	NR
Yu [27]	10	10	40–65	10.6 ± 4.4	11.1 ± 4.2		WHO 1999	Yes	Yes	No	No	NR
Zou and Liu [24]	50	50	50.40 ± 1.92	50.14 ± 1.53	10.87 ± 3.64	11.37 ± 3.58	NR	No	Yes	No	No	NR

E, experimental group; C, control group; ADs, adverse events; NR, no report; ADA, American Diabetes Association; WHO, World Health Organization; IUA, International Union of Angiology; CMA, Chinese medical association.

## Abbreviations

CI: Confidence interval
RCT: Randomized controlled trials
RR: Risk ratios
DFU: Diabetic foot ulcer
KFXL: KangFuXin liquid
E: Experimental group
C: Control group
Ads: Adverse events
NR: No report
ADA: American diabetes association
WHO: World health organization
IUA: International union of angiology
CMA: Chinese medical association.

## Supplementary Materials

Supplementary Materials The supplementary materials that I have included in the manuscript are PRISMA (Preferred Reporting Items for Systematic Reviews and Meta-Analyses) and cover letter. PRISMA is an evidence-based minimum set of items for reporting in systematic reviews and meta-analyses.
